# Supplementary material for: The effects of genital myiasis on the diversity of the vaginal microbiota in female Bactrian camels
Source: BMC Vet Res. 2022 Mar 5;18:87. doi: 10.1186/s12917-022-03189-5 (PMC8897907; doi:10.1186/s12917-022-03189-5)
Supplement: Supplementary file 5 — Additional file 5. [file 12917_2022_3189_MOESM5_ESM.zip › MPL201709200_16s_yy/Treat1/css/js_css/jsPDF-master.zip!/jsPDF-master/docs/plugins_acroform.js.html]

plugins/acroform.js - Documentation


## Home

### Classes

- jsPDF

### Global

- addFont
- addHTML
- addMetadata
- addPage
- autoPrint
- CapJoinStyles
- circle
- ellipse
- getFontList
- lines
- lstext
- output
- rect
- roundedRect
- save
- setDisplayMode
- setDrawColor
- setFillColor
- setFont
- setFontSize
- setFontStyle
- setLineCap
- setLineJoin
- setLineWidth
- setPage
- setProperties
- setTextColor
- text
- triangle

# plugins/acroform.js

```
/**
 * jsPDF AcroForm Plugin
 * Copyright (c) 2016 Alexander Weidt, https://github.com/BiggA94
 *
 * Licensed under the MIT License.
 * http://opensource.org/licenses/mit-license
 */

(window.AcroForm = function (jsPDFAPI) {
    'use strict';
    
    var AcroForm = window.AcroForm;

    AcroForm.scale = function (x) {
        return (x * (acroformPlugin.internal.scaleFactor / 1));// 1 = (96 / 72)
    };
    AcroForm.antiScale = function (x) {
        return ((1 / acroformPlugin.internal.scaleFactor ) * x);
    };

    var acroformPlugin = {
        fields: [],
        xForms: [],
        /**
         * acroFormDictionaryRoot contains information about the AcroForm Dictionary
         * 0: The Event-Token, the AcroFormDictionaryCallback has
         * 1: The Object ID of the Root
         */
        acroFormDictionaryRoot: null,
        /**
         * After the PDF gets evaluated, the reference to the root has to be reset,
         * this indicates, whether the root has already been printed out
         */
        printedOut: false,
        internal: null
    };

    jsPDF.API.acroformPlugin = acroformPlugin;

    var annotReferenceCallback = function () {
        for (var i in this.acroformPlugin.acroFormDictionaryRoot.Fields) {
            var formObject = this.acroformPlugin.acroFormDictionaryRoot.Fields[i];
            // add Annot Reference!
            if (formObject.hasAnnotation) {
                // If theres an Annotation Widget in the Form Object, put the Reference in the /Annot array
                createAnnotationReference.call(this, formObject);
            }
        }
    };

    var createAcroForm = function () {
        if (this.acroformPlugin.acroFormDictionaryRoot) {
            //return;
            throw new Error("Exception while creating AcroformDictionary");
        }

        // The Object Number of the AcroForm Dictionary
        this.acroformPlugin.acroFormDictionaryRoot = new AcroForm.AcroFormDictionary();

        this.acroformPlugin.internal = this.internal;

        // add Callback for creating the AcroForm Dictionary
        this.acroformPlugin.acroFormDictionaryRoot._eventID = this.internal.events.subscribe('postPutResources', AcroFormDictionaryCallback);

        this.internal.events.subscribe('buildDocument', annotReferenceCallback); //buildDocument

        // Register event, that is triggered when the DocumentCatalog is written, in order to add /AcroForm
        this.internal.events.subscribe('putCatalog', putCatalogCallback);

        // Register event, that creates all Fields
        this.internal.events.subscribe('postPutPages', createFieldCallback);
    };

    /**
     * Create the Reference to the widgetAnnotation, so that it gets referenced in the Annot[] int the+
     * (Requires the Annotation Plugin)
     */
    var createAnnotationReference = function (object) {
        var options = {
            type: 'reference',
            object: object
        };
        jsPDF.API.annotationPlugin.annotations[this.internal.getPageInfo(object.page).pageNumber].push(options);
    };

    var putForm = function (formObject) {
        if (this.acroformPlugin.printedOut) {
            this.acroformPlugin.printedOut = false;
            this.acroformPlugin.acroFormDictionaryRoot = null;
        }
        if (!this.acroformPlugin.acroFormDictionaryRoot) {
            createAcroForm.call(this);
        }
        this.acroformPlugin.acroFormDictionaryRoot.Fields.push(formObject);
    };

    // Callbacks

    var putCatalogCallback = function () {
        //Put reference to AcroForm to DocumentCatalog
        if (typeof this.acroformPlugin.acroFormDictionaryRoot != 'undefined') { // for safety, shouldn't normally be the case
            this.internal.write('/AcroForm ' + this.acroformPlugin.acroFormDictionaryRoot.objId + ' '
                + 0 + ' R');
        } else {
            console.log('Root missing...');
        }
    };

    /**
     * Adds /Acroform X 0 R to Document Catalog,
     * and creates the AcroForm Dictionary
     */
    var AcroFormDictionaryCallback = function () {
        // Remove event
        this.internal.events.unsubscribe(this.acroformPlugin.acroFormDictionaryRoot._eventID);

        delete this.acroformPlugin.acroFormDictionaryRoot._eventID;

        this.acroformPlugin.printedOut = true;
    };

    /**
     * Creates the single Fields and writes them into the Document
     *
     * If fieldArray is set, use the fields that are inside it instead of the fields from the AcroRoot
     * (for the FormXObjects...)
     */
    var createFieldCallback = function (fieldArray) {
        var standardFields = (!fieldArray);

        if (!fieldArray) {
            // in case there is no fieldArray specified, we want to print out the Fields of the AcroForm
            // Print out Root
            this.internal.newObjectDeferredBegin(this.acroformPlugin.acroFormDictionaryRoot.objId);
            this.internal.out(this.acroformPlugin.acroFormDictionaryRoot.getString());
        }

        var fieldArray = fieldArray || this.acroformPlugin.acroFormDictionaryRoot.Kids;

        for (var i in fieldArray) {
            var key = i;
            var form = fieldArray[i];

            var oldRect = form.Rect;

            if (form.Rect) {
                form.Rect = AcroForm.internal.calculateCoordinates.call(this, form.Rect);
            }

            // Start Writing the Object
            this.internal.newObjectDeferredBegin(form.objId);

            var content = "";
            content += (form.objId + " 0 obj\n");

            content += ("<<\n" + form.getContent());

            form.Rect = oldRect;

            if (form.hasAppearanceStream && !form.appearanceStreamContent) {
                // Calculate Appearance
                var appearance = AcroForm.internal.calculateAppearanceStream.call(this, form);
                content += "/AP << /N " + appearance + " >>\n";

                this.acroformPlugin.xForms.push(appearance);
            }

            // Assume AppearanceStreamContent is a Array with N,R,D (at least one of them!)
            if (form.appearanceStreamContent) {
                content += "/AP << ";
                // Iterate over N,R and D
                for (var k in form.appearanceStreamContent) {
                    var value = form.appearanceStreamContent[k];
                    content += ("/" + k + " ");
                    content += "<< ";
                    if (Object.keys(value).length >= 1 || Array.isArray(value)) {
                        // appearanceStream is an Array or Object!
                        for (var i in value) {
                            var obj = value[i];
                            if (typeof obj === 'function') {
                                // if Function is referenced, call it in order to get the FormXObject
                                obj = obj.call(this, form);
                            }
                            content += ("/" + i + " " + obj + " ");

                            // In case the XForm is already used, e.g. OffState of CheckBoxes, don't add it
                            if (!(this.acroformPlugin.xForms.indexOf(obj) >= 0))
                                this.acroformPlugin.xForms.push(obj);
                        }
                    } else {
                        var obj = value;
                        if (typeof obj === 'function') {
                            // if Function is referenced, call it in order to get the FormXObject
                            obj = obj.call(this, form);
                        }
                        content += ("/" + i + " " + obj + " \n");
                        if (!(this.acroformPlugin.xForms.indexOf(obj) >= 0))
                            this.acroformPlugin.xForms.push(obj);
                    }
                    content += " >>\n";
                }

                // appearance stream is a normal Object..
                content += (">>\n");
            }

            content += (">>\nendobj\n");

            this.internal.out(content);

        }
        if (standardFields) {
            createXFormObjectCallback.call(this, this.acroformPlugin.xForms);
        }
    };

    var createXFormObjectCallback = function (fieldArray) {
        for (var i in fieldArray) {
            var key = i;
            var form = fieldArray[i];
            // Start Writing the Object
            this.internal.newObjectDeferredBegin(form && form.objId);

            var content = "";
            content += form ? form.getString() : '';
            this.internal.out(content);

            delete fieldArray[key];
        }
    };

    // Public:

    jsPDFAPI.addField = function (fieldObject) {
        //var opt = parseOptions(fieldObject);
        if (fieldObject instanceof AcroForm.TextField) {
            addTextField.call(this, fieldObject);
        } else if (fieldObject instanceof AcroForm.ChoiceField) {
            addChoiceField.call(this, fieldObject);
        } else if (fieldObject instanceof AcroForm.Button) {
            addButton.call(this, fieldObject);
        } else if (fieldObject instanceof AcroForm.ChildClass) {
            putForm.call(this, fieldObject);
        } else if (fieldObject) {
            // try to put..
            putForm.call(this, fieldObject);
        }
        fieldObject.page = this.acroformPlugin.internal.getCurrentPageInfo().pageNumber;
        return this;
    };


    // ############### sort in:

    /**
     * Button
     * FT = Btn
     */
    var addButton = function (options) {
        var options = options || new AcroForm.Field();

        options.FT = '/Btn';

        /**
         * Calculating the Ff entry:
         *
         * The Ff entry contains flags, that have to be set bitwise
         * In the Following the number in the Comment is the BitPosition
         */
        var flags = options.Ff || 0;

        // 17, Pushbutton
        if (options.pushbutton) {
            // Options.pushbutton should be 1 or 0
            flags = AcroForm.internal.setBitPosition(flags, 17);
            delete options.pushbutton;
        }

        //16, Radio
        if (options.radio) {
            //flags = options.Ff | options.radio << 15;
            flags = AcroForm.internal.setBitPosition(flags, 16);
            delete options.radio;
        }

        // 15, NoToggleToOff (Radio buttons only
        if (options.noToggleToOff) {
            //flags = options.Ff | options.noToggleToOff << 14;
            flags = AcroForm.internal.setBitPosition(flags, 15);
            //delete options.noToggleToOff;
        }

        // In case, there is no Flag set, it is a check-box
        options.Ff = flags;

        putForm.call(this, options);

    };


    var addTextField = function (options) {
        var options = options || new AcroForm.Field();

        options.FT = '/Tx';

        /**
         * Calculating the Ff entry:
         *
         * The Ff entry contains flags, that have to be set bitwise
         * In the Following the number in the Comment is the BitPosition
         */

        var flags = options.Ff || 0;

        // 13, multiline
        if (options.multiline) {
            // Set Flag
            flags = flags | (1 << 12);
            // Remove multiline from FieldObject
            //delete options.multiline;
        }

        // 14, Password
        if (options.password) {
            flags = flags | (1 << 13);
            //delete options.password;
        }

        // 21, FileSelect, PDF 1.4...
        if (options.fileSelect) {
            flags = flags | (1 << 20);
            //delete options.fileSelect;
        }

        // 23, DoNotSpellCheck, PDF 1.4...
        if (options.doNotSpellCheck) {
            flags = flags | (1 << 22);
            //delete options.doNotSpellCheck;
        }

        // 24, DoNotScroll, PDF 1.4...
        if (options.doNotScroll) {
            flags = flags | (1 << 23);
            //delete options.doNotScroll;
        }

        options.Ff = options.Ff || flags;

        // Add field
        putForm.call(this, options);
    };

    var addChoiceField = function (opt) {
        var options = opt || new AcroForm.Field();

        options.FT = '/Ch';

        /**
         * Calculating the Ff entry:
         *
         * The Ff entry contains flags, that have to be set bitwise
         * In the Following the number in the Comment is the BitPosition
         */

        var flags = options.Ff || 0;

        // 18, Combo (If not set, the choiceField is a listBox!!)
        if (options.combo) {
            // Set Flag
            flags = AcroForm.internal.setBitPosition(flags, 18);
            // Remove combo from FieldObject
            delete options.combo;
        }

        // 19, Edit
        if (options.edit) {
            flags = AcroForm.internal.setBitPosition(flags, 19);
            delete options.edit;
        }

        // 20, Sort
        if (options.sort) {
            flags = AcroForm.internal.setBitPosition(flags, 20);
            delete options.sort;
        }

        // 22, MultiSelect (PDF 1.4)
        if (options.multiSelect && this.internal.getPDFVersion() >= 1.4) {
            flags = AcroForm.internal.setBitPosition(flags, 22);
            delete options.multiSelect;
        }

        // 23, DoNotSpellCheck (PDF 1.4)
        if (options.doNotSpellCheck && this.internal.getPDFVersion() >= 1.4) {
            flags = AcroForm.internal.setBitPosition(flags, 23);
            delete options.doNotSpellCheck;
        }

        options.Ff = flags;

        //options.hasAnnotation = true;

        // Add field
        putForm.call(this, options);
    };
})(jsPDF.API);

var AcroForm = window.AcroForm;

AcroForm.internal = {};

AcroForm.createFormXObject = function (formObject) {
    var xobj = new AcroForm.FormXObject;
    var height = AcroForm.Appearance.internal.getHeight(formObject) || 0;
    var width = AcroForm.Appearance.internal.getWidth(formObject) || 0;
    xobj.BBox = [0, 0, width, height];
    return xobj;
};

// Contains Methods for creating standard appearances
AcroForm.Appearance = {
    CheckBox: {
        createAppearanceStream: function () {
            var appearance = {
                N: {
                    On: AcroForm.Appearance.CheckBox.YesNormal
                },
                D: {
                    On: AcroForm.Appearance.CheckBox.YesPushDown,
                    Off: AcroForm.Appearance.CheckBox.OffPushDown
                }
            };

            return appearance;
        },
        /**
         * If any other icons are needed, the number between the brackets can be changed
         * @returns {string}
         */
        createMK: function () {
            // 3-> Hook
            return "<< /CA (3)>>";
        },
        /**
         * Returns the standard On Appearance for a CheckBox
         * @returns {AcroForm.FormXObject}
         */
        YesPushDown: function (formObject) {
            var xobj = AcroForm.createFormXObject(formObject);
            var stream = "";
            // F13 is ZapfDingbats (Symbolic)
            formObject.Q = 1; // set text-alignment as centered
            var calcRes = AcroForm.internal.calculateX(formObject, "3", "ZapfDingbats", 50);
            stream += "0.749023 g\n\
             0 0 " + AcroForm.Appearance.internal.getWidth(formObject) + " " + AcroForm.Appearance.internal.getHeight(formObject) + " re\n\
             f\n\
             BMC\n\
             q\n\
             0 0 1 rg\n\
             /F13 " + calcRes.fontSize + " Tf 0 g\n\
             BT\n";
            stream += calcRes.text;
            stream += "ET\n\
             Q\n\
             EMC\n";
            xobj.stream = stream;
            return xobj;
        },

        YesNormal: function (formObject) {
            var xobj = AcroForm.createFormXObject(formObject);
            var stream = "";
            formObject.Q = 1; // set text-alignment as centered
            var calcRes = AcroForm.internal.calculateX(formObject, "3", "ZapfDingbats", AcroForm.Appearance.internal.getHeight(formObject) * 0.9);
            stream += "1 g\n\
0 0 " + AcroForm.Appearance.internal.getWidth(formObject) + " " + AcroForm.Appearance.internal.getHeight(formObject) + " re\n\
f\n\
q\n\
0 0 1 rg\n\
0 0 " + (AcroForm.Appearance.internal.getWidth(formObject) - 1) + " " + (AcroForm.Appearance.internal.getHeight(formObject) - 1) + " re\n\
W\n\
n\n\
0 g\n\
BT\n\
/F13 " + calcRes.fontSize + " Tf 0 g\n";
            stream += calcRes.text;
            stream += "ET\n\
             Q\n";
            xobj.stream = stream;
            return xobj;
        },

        /**
         * Returns the standard Off Appearance for a CheckBox
         * @returns {AcroForm.FormXObject}
         */
        OffPushDown: function (formObject) {
            var xobj = AcroForm.createFormXObject(formObject);
            var stream = "";
            stream += "0.749023 g\n\
            0 0 " + AcroForm.Appearance.internal.getWidth(formObject) + " " + AcroForm.Appearance.internal.getHeight(formObject) + " re\n\
            f\n";
            xobj.stream = stream;
            return xobj;
        }
    },

    RadioButton: {
        Circle: {
            createAppearanceStream: function (name) {
                var appearanceStreamContent = {
                    D: {
                        'Off': AcroForm.Appearance.RadioButton.Circle.OffPushDown
                    },
                    N: {}
                };
                appearanceStreamContent.N[name] = AcroForm.Appearance.RadioButton.Circle.YesNormal;
                appearanceStreamContent.D[name] = AcroForm.Appearance.RadioButton.Circle.YesPushDown;
                return appearanceStreamContent;
            },
            createMK: function () {
                return "<< /CA (l)>>";
            },

            YesNormal: function (formObject) {
                var xobj = AcroForm.createFormXObject(formObject);
                var stream = "";
                // Make the Radius of the Circle relative to min(height, width) of formObject
                var DotRadius = (AcroForm.Appearance.internal.getWidth(formObject) <= AcroForm.Appearance.internal.getHeight(formObject)) ?
                AcroForm.Appearance.internal.getWidth(formObject) / 4 : AcroForm.Appearance.internal.getHeight(formObject) / 4;
                // The Borderpadding...
                DotRadius *= 0.9;
                var c = AcroForm.Appearance.internal.Bezier_C;
                /*
                 The Following is a Circle created with Bezier-Curves.
                 */
                stream += "q\n\
1 0 0 1 " + AcroForm.Appearance.internal.getWidth(formObject) / 2 + " " + AcroForm.Appearance.internal.getHeight(formObject) / 2 + " cm\n\
" + DotRadius + " 0 m\n\
" + DotRadius + " " + DotRadius * c + " " + DotRadius * c + " " + DotRadius + " 0 " + DotRadius + " c\n\
-" + DotRadius * c + " " + DotRadius + " -" + DotRadius + " " + DotRadius * c + " -" + DotRadius + " 0 c\n\
-" + DotRadius + " -" + DotRadius * c + " -" + DotRadius * c + " -" + DotRadius + " 0 -" + DotRadius + " c\n\
" + DotRadius * c + " -" + DotRadius + " " + DotRadius + " -" + DotRadius * c + " " + DotRadius + " 0 c\n\
f\n\
Q\n";
                xobj.stream = stream;
                return xobj;
            },
            YesPushDown: function (formObject) {
                var xobj = AcroForm.createFormXObject(formObject);
                var stream = "";
                var DotRadius = (AcroForm.Appearance.internal.getWidth(formObject) <= AcroForm.Appearance.internal.getHeight(formObject)) ?
                AcroForm.Appearance.internal.getWidth(formObject) / 4 : AcroForm.Appearance.internal.getHeight(formObject) / 4;
                // The Borderpadding...
                DotRadius *= 0.9;
                // Save results for later use; no need to waste processor ticks on doing math
                var k = DotRadius * 2;
                // var c = AcroForm.Appearance.internal.Bezier_C;
                var kc = k * AcroForm.Appearance.internal.Bezier_C;
                var dc = DotRadius * AcroForm.Appearance.internal.Bezier_C;
//                 stream += "0.749023 g\n\
//             q\n\
//           1 0 0 1 " + AcroForm.Appearance.internal.getWidth(formObject) / 2 + " " + AcroForm.Appearance.internal.getHeight(formObject) / 2 + " cm\n\
// " + DotRadius * 2 + " 0 m\n\
// " + DotRadius * 2 + " " + DotRadius * 2 * c + " " + DotRadius * 2 * c + " " + DotRadius * 2 + " 0 " + DotRadius * 2 + " c\n\
// -" + DotRadius * 2 * c + " " + DotRadius * 2 + " -" + DotRadius * 2 + " " + DotRadius * 2 * c + " -" + DotRadius * 2 + " 0 c\n\
// -" + DotRadius * 2 + " -" + DotRadius * 2 * c + " -" + DotRadius * 2 * c + " -" + DotRadius * 2 + " 0 -" + DotRadius * 2 + " c\n\
// " + DotRadius * 2 * c + " -" + DotRadius * 2 + " " + DotRadius * 2 + " -" + DotRadius * 2 * c + " " + DotRadius * 2 + " 0 c\n\
//             f\n\
//             Q\n\
//             0 g\n\
//             q\n\
//             1 0 0 1 " + AcroForm.Appearance.internal.getWidth(formObject) / 2 + " " + AcroForm.Appearance.internal.getHeight(formObject) / 2 + " cm\n\
// " + DotRadius + " 0 m\n\
// " + DotRadius + " " + DotRadius * c + " " + DotRadius * c + " " + DotRadius + " 0 " + DotRadius + " c\n\
// -" + DotRadius * c + " " + DotRadius + " -" + DotRadius + " " + DotRadius * c + " -" + DotRadius + " 0 c\n\
// -" + DotRadius + " -" + DotRadius * c + " -" + DotRadius * c + " -" + DotRadius + " 0 -" + DotRadius + " c\n\
// " + DotRadius * c + " -" + DotRadius + " " + DotRadius + " -" + DotRadius * c + " " + DotRadius + " 0 c\n\
//             f\n\
//             Q\n";

//  FASTER VERSION with less processor ticks spent on math operations
                stream += "0.749023 g\n\
            q\n\
           1 0 0 1 " + AcroForm.Appearance.internal.getWidth(formObject) / 2 + " " + AcroForm.Appearance.internal.getHeight(formObject) / 2 + " cm\n\
" + k + " 0 m\n\
" + k + " " + kc + " " + kc + " " + k + " 0 " + k + " c\n\
-" + kc + " " + k + " -" + k + " " + kc + " -" + k + " 0 c\n\
-" + k + " -" + kc + " -" + kc + " -" + k + " 0 -" + k + " c\n\
" + kc + " -" + k + " " + k + " -" + kc + " " + k + " 0 c\n\
            f\n\
            Q\n\
            0 g\n\
            q\n\
            1 0 0 1 " + AcroForm.Appearance.internal.getWidth(formObject) / 2 + " " + AcroForm.Appearance.internal.getHeight(formObject) / 2 + " cm\n\
" + DotRadius + " 0 m\n\
" + DotRadius + " " + dc + " " + dc + " " + DotRadius + " 0 " + DotRadius + " c\n\
-" + dc + " " + DotRadius + " -" + DotRadius + " " + dc + " -" + DotRadius + " 0 c\n\
-" + DotRadius + " -" + dc + " -" + dc + " -" + DotRadius + " 0 -" + DotRadius + " c\n\
" + dc + " -" + DotRadius + " " + DotRadius + " -" + dc + " " + DotRadius + " 0 c\n\
            f\n\
            Q\n";
                xobj.stream = stream;
                return xobj;
            },
            OffPushDown: function (formObject) {
                var xobj = AcroForm.createFormXObject(formObject);
                var stream = "";
                var DotRadius = (AcroForm.Appearance.internal.getWidth(formObject) <= AcroForm.Appearance.internal.getHeight(formObject)) ?
                AcroForm.Appearance.internal.getWidth(formObject) / 4 : AcroForm.Appearance.internal.getHeight(formObject) / 4;
                // The Borderpadding...
                DotRadius *= 0.9;
                // Save results for later use; no need to waste processor ticks on doing math
                var k = DotRadius * 2;
                // var c = AcroForm.Appearance.internal.Bezier_C;
                var kc = k * AcroForm.Appearance.internal.Bezier_C;
//                 stream += "0.749023 g\n\
//             q\n\
//  1 0 0 1 " + AcroForm.Appearance.internal.getWidth(formObject) / 2 + " " + AcroForm.Appearance.internal.getHeight(formObject) / 2 + " cm\n\
// " + DotRadius * 2 + " 0 m\n\
// " + DotRadius * 2 + " " + DotRadius * 2 * c + " " + DotRadius * 2 * c + " " + DotRadius * 2 + " 0 " + DotRadius * 2 + " c\n\
// -" + DotRadius * 2 * c + " " + DotRadius * 2 + " -" + DotRadius * 2 + " " + DotRadius * 2 * c + " -" + DotRadius * 2 + " 0 c\n\
// -" + DotRadius * 2 + " -" + DotRadius * 2 * c + " -" + DotRadius * 2 * c + " -" + DotRadius * 2 + " 0 -" + DotRadius * 2 + " c\n\
// " + DotRadius * 2 * c + " -" + DotRadius * 2 + " " + DotRadius * 2 + " -" + DotRadius * 2 * c + " " + DotRadius * 2 + " 0 c\n\
//             f\n\
//             Q\n";

//  FASTER VERSION with less processor ticks spent on math operations
                stream += "0.749023 g\n\
            q\n\
 1 0 0 1 " + AcroForm.Appearance.internal.getWidth(formObject) / 2 + " " + AcroForm.Appearance.internal.getHeight(formObject) / 2 + " cm\n\
" + k + " 0 m\n\
" + k + " " + kc + " " + kc + " " + k + " 0 " + k + " c\n\
-" + kc + " " + k + " -" + k + " " + kc + " -" + k + " 0 c\n\
-" + k + " -" + kc + " -" + kc + " -" + k + " 0 -" + k + " c\n\
" + kc + " -" + k + " " + k + " -" + kc + " " + k + " 0 c\n\
            f\n\
            Q\n";
                xobj.stream = stream;
                return xobj;
            },
        },

        Cross: {
            /**
             * Creates the Actual AppearanceDictionary-References
             * @param name
             * @returns
             */
            createAppearanceStream: function (name) {
                var appearanceStreamContent = {
                    D: {
                        'Off': AcroForm.Appearance.RadioButton.Cross.OffPushDown
                    },
                    N: {}
                };
                appearanceStreamContent.N[name] = AcroForm.Appearance.RadioButton.Cross.YesNormal;
                appearanceStreamContent.D[name] = AcroForm.Appearance.RadioButton.Cross.YesPushDown;
                return appearanceStreamContent;
            },
            createMK: function () {
                return "<< /CA (8)>>";
            },


            YesNormal: function (formObject) {
                var xobj = AcroForm.createFormXObject(formObject);
                var stream = "";
                var cross = AcroForm.Appearance.internal.calculateCross(formObject);
                stream += "q\n\
            1 1 " + (AcroForm.Appearance.internal.getWidth(formObject) - 2) + " " + (AcroForm.Appearance.internal.getHeight(formObject) - 2) + " re\n\
            W\n\
            n\n\
            " + cross.x1.x + " " + cross.x1.y + " m\n\
            " + cross.x2.x + " " + cross.x2.y + " l\n\
            " + cross.x4.x + " " + cross.x4.y + " m\n\
            " + cross.x3.x + " " + cross.x3.y + " l\n\
            s\n\
            Q\n";
                xobj.stream = stream;
                return xobj;
            },
            YesPushDown: function (formObject) {
                var xobj = AcroForm.createFormXObject(formObject);
                var cross = AcroForm.Appearance.internal.calculateCross(formObject);
                var stream = "";
                stream += "0.749023 g\n\
            0 0 " + AcroForm.Appearance.internal.getWidth(formObject) + " " + AcroForm.Appearance.internal.getHeight(formObject) + " re\n\
            f\n\
            q\n\
            1 1 " + (AcroForm.Appearance.internal.getWidth(formObject) - 2) + " " + (AcroForm.Appearance.internal.getHeight(formObject) - 2) + " re\n\
            W\n\
            n\n\
            " + cross.x1.x + " " + cross.x1.y + " m\n\
            " + cross.x2.x + " " + cross.x2.y + " l\n\
            " + cross.x4.x + " " + cross.x4.y + " m\n\
            " + cross.x3.x + " " + cross.x3.y + " l\n\
            s\n\
            Q\n";
                xobj.stream = stream;
                return xobj;
            },
            OffPushDown: function (formObject) {
                var xobj = AcroForm.createFormXObject(formObject);
                var stream = "";
                stream += "0.749023 g\n\
            0 0 " + AcroForm.Appearance.internal.getWidth(formObject) + " " + AcroForm.Appearance.internal.getHeight(formObject) + " re\n\
            f\n";
                xobj.stream = stream;
                return xobj;
            }
        },
    },

    /**
     * Returns the standard Appearance
     * @returns {AcroForm.FormXObject}
     */
    createDefaultAppearanceStream: function (formObject) {
        var stream = "";
        // Set Helvetica to Standard Font (size: auto)
        // Color: Black
        stream += "/Helv 0 Tf 0 g";
        return stream;
    }
};

AcroForm.Appearance.internal = {
    Bezier_C: 0.551915024494,

    calculateCross: function (formObject) {
        var min = function (x, y) {
            return (x > y) ? y : x;
        };

        var width = AcroForm.Appearance.internal.getWidth(formObject);
        var height = AcroForm.Appearance.internal.getHeight(formObject);
        var a = min(width, height);
        var crossSize = a;
        var borderPadding = 2; // The Padding in px


        var cross = {
            x1: { // upperLeft
                x: (width - a) / 2,
                y: ((height - a) / 2) + a,//height - borderPadding
            },
            x2: { // lowerRight
                x: ((width - a) / 2) + a,
                y: ((height - a) / 2)//borderPadding
            },
            x3: { // lowerLeft
                x: (width - a) / 2,
                y: ((height - a) / 2)//borderPadding
            },
            x4: { // upperRight
                x: ((width - a) / 2) + a,
                y: ((height - a) / 2) + a,//height - borderPadding
            }
        };

        return cross;
    },
};
AcroForm.Appearance.internal.getWidth = function (formObject) {
    return formObject.Rect[2];//(formObject.Rect[2] - formObject.Rect[0]) || 0;
};
AcroForm.Appearance.internal.getHeight = function (formObject) {
    return formObject.Rect[3];//(formObject.Rect[1] - formObject.Rect[3]) || 0;
};

// ##########################

//### For inheritance:
AcroForm.internal.inherit = function (child, parent) {
    var ObjectCreate = Object.create || function (o) {
            var F = function () {
            };
            F.prototype = o;
            return new F();
        };
    child.prototype = Object.create(parent.prototype);
    child.prototype.constructor = child;
};

// ### Handy Functions:

AcroForm.internal.arrayToPdfArray = function (array) {
    if (Array.isArray(array)) {
        var content = ' [';
        for (var i in array) {
            var element = array[i].toString();
            content += element;
            content += ((i < array.length - 1) ? ' ' : '');
        }
        content += ']';

        return content;
    }
};

AcroForm.internal.toPdfString = function (string) {
    string = string || "";

    // put Bracket at the Beginning of the String
    if (string.indexOf('(') !== 0) {
        string = '(' + string;
    }
    
    if (string.substring(string.length - 1) != ')') {
        string += '(';
    }
    return string;
};

// ##########################
//          Classes
// ##########################


AcroForm.PDFObject = function () {
    // The Object ID in the PDF Object Model
    // todo
    var _objId;
    Object.defineProperty(this, 'objId', {
        get: function () {
            if (!_objId) {
                if (this.internal) {
                    _objId = this.internal.newObjectDeferred();
                } else if (jsPDF.API.acroformPlugin.internal) {
                    // todo - find better option, that doesn't rely on a Global Static var
                    _objId = jsPDF.API.acroformPlugin.internal.newObjectDeferred();
                }
            }
            if (!_objId) {
                console.log("Couldn't create Object ID");
            }
            return _objId
        },
        configurable: false
    });
};

AcroForm.PDFObject.prototype.toString = function () {
    return this.objId + " 0 R";
};

AcroForm.PDFObject.prototype.getString = function () {
    var res = this.objId + " 0 obj\n<<";
    var content = this.getContent();

    res += content + ">>\n";
    if (this.stream) {
        res += "stream\n";
        res += this.stream;
        res += "endstream\n";
    }
    res += "endobj\n";
    return res;
};

AcroForm.PDFObject.prototype.getContent = function () {
    /**
     * Prints out all enumerable Variables from the Object
     * @param fieldObject
     * @returns {string}
     */
    var createContentFromFieldObject = function (fieldObject) {
        var content = '';

        var keys = Object.keys(fieldObject).filter(function (key) {
            return (key != 'content' && key != 'appearanceStreamContent' && key.substring(0, 1) != "_");
        });

        for (var i in keys) {
            var key = keys[i];
            var value = fieldObject[key];

            /*if (key == 'Rect' && value) {
             value = AcroForm.internal.calculateCoordinates.call(jsPDF.API.acroformPlugin.internal, value);
             }*/

            if (value) {
                if (Array.isArray(value)) {
                    content += '/' + key + ' ' + AcroForm.internal.arrayToPdfArray(value) + "\n";
                } else if (value instanceof AcroForm.PDFObject) {
                    // In case it is a reference to another PDFObject, take the referennce number
                    content += '/' + key + ' ' + value.objId + " 0 R" + "\n";
                } else {
                    content += '/' + key + ' ' + value + '\n';
                }
            }
        }
        return content;
    };

    var object = "";

    object += createContentFromFieldObject(this);
    return object;
};

AcroForm.FormXObject = function () {
    AcroForm.PDFObject.call(this);
    this.Type = "/XObject";
    this.Subtype = "/Form";
    this.FormType = 1;
    this.BBox;
    this.Matrix;
    this.Resources = "2 0 R";
    this.PieceInfo;
    var _stream;
    Object.defineProperty(this, 'Length', {
        enumerable: true,
        get: function () {
            return (_stream !== undefined) ? _stream.length : 0;
        }
    });
    Object.defineProperty(this, 'stream', {
        enumerable: false,
        set: function (val) {
            _stream = val;
        },
        get: function () {
            if (_stream) {
                return _stream;
            } else {
                return null;
            }
        }
    });
};

AcroForm.internal.inherit(AcroForm.FormXObject, AcroForm.PDFObject);

AcroForm.AcroFormDictionary = function () {
    AcroForm.PDFObject.call(this);
    var _Kids = [];
    Object.defineProperty(this, 'Kids', {
        enumerable: false,
        configurable: true,
        get: function () {
            if (_Kids.length > 0) {
                return _Kids;
            } else {
                return;
            }
        }
    });
    Object.defineProperty(this, 'Fields', {
        enumerable: true,
        configurable: true,
        get: function () {
            return _Kids;
        }
    });
    // Default Appearance
    this.DA;
};

AcroForm.internal.inherit(AcroForm.AcroFormDictionary, AcroForm.PDFObject);


// ##### The Objects, the User can Create:


// The Field Object contains the Variables, that every Field needs
// Rectangle for Appearance: lower_left_X, lower_left_Y, width, height
AcroForm.Field = function () {
    'use strict';
    AcroForm.PDFObject.call(this);

    var _Rect;
    Object.defineProperty(this, 'Rect', {
        enumerable: true,
        configurable: false,
        get: function () {
            if (!_Rect) {
                return;
            }
            var tmp = _Rect;
            //var calculatedRes = AcroForm.internal.calculateCoordinates(_Rect); // do later!
            return tmp
        },
        set: function (val) {
            _Rect = val;
        }
    });

    var _FT = "";
    Object.defineProperty(this, 'FT', {
        enumerable: true,
        set: function (val) {
            _FT = val
        },
        get: function () {
            return _FT
        }
    });
    /**
     * The Partial name of the Field Object.
     * It has to be unique.
     */
    var _T;

    Object.defineProperty(this, 'T', {
        enumerable: true,
        configurable: false,
        set: function (val) {
            _T = val;
        },
        get: function () {
            if (!_T || _T.length < 1) {
                if (this instanceof AcroForm.ChildClass) {
                    // In case of a Child from a Radio´Group, you don't need a FieldName!!!
                    return;
                }
                return "(FieldObject" + (AcroForm.Field.FieldNum++) + ")";
            }
            if (_T.substring(0, 1) == "(" && _T.substring(_T.length - 1)) {
                return _T;
            }
            return "(" + _T + ")";
        }
    });

    var _DA;
    // Defines the default appearance (Needed for variable Text)
    Object.defineProperty(this, 'DA', {
        enumerable: true,
        get: function () {
            if (!_DA) {
                return;
            }
            return '(' + _DA + ')'
        },
        set: function (val) {
            _DA = val
        }
    });

    var _DV;
    // Defines the default value
    Object.defineProperty(this, 'DV', {
        enumerable: true,
        configurable: true,
        get: function () {
            if (!_DV) {
                return;
            }
            return _DV
        },
        set: function (val) {
            _DV = val
        }
    });

    //this.Type = "/Annot";
    //this.Subtype = "/Widget";
    Object.defineProperty(this, 'Type', {
        enumerable: true,
        get: function () {
            return (this.hasAnnotation) ? "/Annot" : null;
        }
    });

    Object.defineProperty(this, 'Subtype', {
        enumerable: true,
        get: function () {
            return (this.hasAnnotation) ? "/Widget" : null;
        }
    });

    /**
     *
     * @type {Array}
     */
    this.BG;

    Object.defineProperty(this, 'hasAnnotation', {
        enumerable: false,
        get: function () {
            if (this.Rect || this.BC || this.BG) {
                return true
            }
            return false;
        }
    });

    Object.defineProperty(this, 'hasAppearanceStream', {
        enumerable: false,
        configurable: true,
        writable: true
    });

    Object.defineProperty(this, 'page', {
        enumerable: false,
        configurable: true,
        writable: true
    });
};
AcroForm.Field.FieldNum = 0;

AcroForm.internal.inherit(AcroForm.Field, AcroForm.PDFObject);

AcroForm.ChoiceField = function () {
    AcroForm.Field.call(this);
    // Field Type = Choice Field
    this.FT = "/Ch";
    // options
    this.Opt = [];
    this.V = '()';
    // Top Index
    this.TI = 0;
    /**
     * Defines, whether the
     * @type {boolean}
     */
    this.combo = false;
    /**
     * Defines, whether the Choice Field is an Edit Field.
     * An Edit Field is automatically an Combo Field.
     */
    Object.defineProperty(this, 'edit', {
        enumerable: true,
        set: function (val) {
            if (val == true) {
                this._edit = true;
                // ComboBox has to be true
                this.combo = true;
            } else {
                this._edit = false;
            }
        },
        get: function () {
            if (!this._edit) {
                return false;
            }
            return this._edit;
        },
        configurable: false
    });
    this.hasAppearanceStream = true;
    Object.defineProperty(this, 'V', {
        get: function() {
            AcroForm.internal.toPdfString();
        }
    });
};
AcroForm.internal.inherit(AcroForm.ChoiceField, AcroForm.Field);
window["ChoiceField"] = AcroForm.ChoiceField;

AcroForm.ListBox = function () {
    AcroForm.ChoiceField.call(this);
    //var combo = true;
};
AcroForm.internal.inherit(AcroForm.ListBox, AcroForm.ChoiceField);
window["ListBox"] = AcroForm.ListBox;

AcroForm.ComboBox = function () {
    AcroForm.ListBox.call(this);
    this.combo = true;
};
AcroForm.internal.inherit(AcroForm.ComboBox, AcroForm.ListBox);
window["ComboBox"] = AcroForm.ComboBox;

AcroForm.EditBox = function () {
    AcroForm.ComboBox.call(this);
    this.edit = true;
};
AcroForm.internal.inherit(AcroForm.EditBox, AcroForm.ComboBox);
window["EditBox"] = AcroForm.EditBox;


AcroForm.Button = function () {
    AcroForm.Field.call(this);
    this.FT = "/Btn";
    //this.hasAnnotation = true;
};
AcroForm.internal.inherit(AcroForm.Button, AcroForm.Field);
window["Button"] = AcroForm.Button;

AcroForm.PushButton = function () {
    AcroForm.Button.call(this);
    this.pushbutton = true;
};
AcroForm.internal.inherit(AcroForm.PushButton, AcroForm.Button);
window["PushButton"] = AcroForm.PushButton;

AcroForm.RadioButton = function () {
    AcroForm.Button.call(this);
    this.radio = true;
    var _Kids = [];
    Object.defineProperty(this, 'Kids', {
        enumerable: true,
        get: function () {
            if (_Kids.length > 0) {
                return _Kids;
            }
        }
    });

    Object.defineProperty(this, '__Kids', {
        get: function () {
            return _Kids;
        }
    });

    var _noToggleToOff;

    Object.defineProperty(this, 'noToggleToOff', {
        enumerable: false,
        get: function () {
            return _noToggleToOff
        },
        set: function (val) {
            _noToggleToOff = val
        }
    });


    //this.hasAnnotation = false;
};
AcroForm.internal.inherit(AcroForm.RadioButton, AcroForm.Button);
window["RadioButton"] = AcroForm.RadioButton;

/*
 * The Child classs of a RadioButton (the radioGroup)
 * -> The single Buttons
 */
AcroForm.ChildClass = function (parent, name) {
    AcroForm.Field.call(this);
    this.Parent = parent;

    // todo: set AppearanceType as variable that can be set from the outside...
    this._AppearanceType = AcroForm.Appearance.RadioButton.Circle; // The Default appearanceType is the Circle
    this.appearanceStreamContent = this._AppearanceType.createAppearanceStream(name);

    // Set Print in the Annot Flag
    this.F = AcroForm.internal.setBitPosition(this.F, 3, 1);

    // Set AppearanceCharacteristicsDictionary with default appearance if field is not interacting with user
    this.MK = this._AppearanceType.createMK(); // (8) -> Cross, (1)-> Circle, ()-> nothing

    // Default Appearance is Off
    this.AS = "/Off";// + name;

    this._Name = name;
};
AcroForm.internal.inherit(AcroForm.ChildClass, AcroForm.Field);

AcroForm.RadioButton.prototype.setAppearance = function (appearance) {
    if (!('createAppearanceStream' in appearance && 'createMK' in appearance)) {
        console.log("Couldn't assign Appearance to RadioButton. Appearance was Invalid!");
        return;
    }
    for (var i in this.__Kids) {
        var child = this.__Kids[i];

        child.appearanceStreamContent = appearance.createAppearanceStream(child._Name);
        child.MK = appearance.createMK();
    }
};

AcroForm.RadioButton.prototype.createOption = function (name) {
    var parent = this;
    var kidCount = this.__Kids.length;

    // Create new Child for RadioGroup
    var child = new AcroForm.ChildClass(parent, name);
    // Add to Parent
    this.__Kids.push(child);

    jsPDF.API.addField(child);

    return child;
};


AcroForm.CheckBox = function () {
    Button.call(this);
    this.appearanceStreamContent = AcroForm.Appearance.CheckBox.createAppearanceStream();
    this.MK = AcroForm.Appearance.CheckBox.createMK();
    this.AS = "/On";
    this.V = "/On";
};
AcroForm.internal.inherit(AcroForm.CheckBox, AcroForm.Button);
window["CheckBox"] = AcroForm.CheckBox;

AcroForm.TextField = function () {
    AcroForm.Field.call(this);
    this.DA = AcroForm.Appearance.createDefaultAppearanceStream();
    this.F = 4;
    var _V;
    Object.defineProperty(this, 'V', {
        get: function () {
            if (_V) {
                return "(" + _V + ")"
            }
            else {
                return _V
            }
        },
        enumerable: true,
        set: function (val) {
            _V = val
        }
    });

    var _DV;
    Object.defineProperty(this, 'DV', {
        get: function () {
            if (_DV) {
                return "(" + _DV + ")"
            }
            else {
                return _DV
            }
        },
        enumerable: true,
        set: function (val) {
            _DV = val
        }
    });

    var _multiline = false;
    Object.defineProperty(this, 'multiline', {
        enumerable: false,
        get: function () {
            return _multiline
        },
        set: function (val) {
            _multiline = val;
        }
    });

    //this.multiline = false;
    //this.password = false;
    /**
     * For PDF 1.4
     * @type {boolean}
     */
    //this.fileSelect = false;
    /**
     * For PDF 1.4
     * @type {boolean}
     */
    //this.doNotSpellCheck = false;
    /**
     * For PDF 1.4
     * @type {boolean}
     */
        //this.doNotScroll = false;

    var _MaxLen = false;
    Object.defineProperty(this, 'MaxLen', {
        enumerable: true,
        get: function () {
            return _MaxLen;
        },
        set: function (val) {
            _MaxLen = val;
        }
    });

    Object.defineProperty(this, 'hasAppearanceStream', {
        enumerable: false,
        get: function () {
            return (this.V || this.DV);
        }
    });

};
AcroForm.internal.inherit(AcroForm.TextField, AcroForm.Field);
window["TextField"] = AcroForm.TextField;

AcroForm.PasswordField = function () {
    TextField.call(this);
    Object.defineProperty(this, 'password', {
        value: true,
        enumerable: false,
        configurable: false,
        writable: false
    });
};
AcroForm.internal.inherit(AcroForm.PasswordField, AcroForm.TextField);
window["PasswordField"] = AcroForm.PasswordField;

// ############ internal functions

/*
 * small workaround for calculating the TextMetric approximately
 * @param text
 * @param fontsize
 * @returns {TextMetrics} (Has Height and Width)
 */
AcroForm.internal.calculateFontSpace = function (text, fontsize, fonttype) {
    var fonttype = fonttype || "helvetica";
    //re-use canvas object for speed improvements
    var canvas = AcroForm.internal.calculateFontSpace.canvas || (AcroForm.internal.calculateFontSpace.canvas = document.createElement('canvas'));

    var context = canvas.getContext('2d');
    context.save();
    var newFont = (fontsize + " " + fonttype);
    context.font = newFont;
    var res = context.measureText(text);
    context.fontcolor = 'black';
    // Calculate height:
    var context = canvas.getContext('2d');
    res.height = context.measureText("3").width * 1.5; // 3 because in ZapfDingbats its a Hook and a 3 in normal fonts
    context.restore();

    var width = res.width;

    return res;
};

AcroForm.internal.calculateX = function (formObject, text, font, maxFontSize) {
    var maxFontSize = maxFontSize || 12;
    var font = font || "helvetica";
    var returnValue = {
        text: "",
        fontSize: ""
    };
    // Remove Brackets
    text = (text.substr(0, 1) == '(') ? text.substr(1) : text;
    text = (text.substr(text.length - 1) == ')') ? text.substr(0, text.length - 1) : text;
    // split into array of words
    var textSplit = text.split(' ');

    /**
     * the color could be ((alpha)||(r,g,b)||(c,m,y,k))
     * @type {string}
     */
    var color = "0 g\n";
    var fontSize = maxFontSize; // The Starting fontSize (The Maximum)
    var lineSpacing = 2;
    var borderPadding = 2;


    var height = AcroForm.Appearance.internal.getHeight(formObject) || 0;
    height = (height < 0) ? -height : height;
    var width = AcroForm.Appearance.internal.getWidth(formObject) || 0;
    width = (width < 0) ? -width : width;

    var isSmallerThanWidth = function (i, lastLine, fontSize) {
        if (i + 1 < textSplit.length) {
            var tmp = lastLine + " " + textSplit[i + 1];
            var TextWidth = ((AcroForm.internal.calculateFontSpace(tmp, fontSize + "px", font).width));
            var FieldWidth = (width - 2 * borderPadding);
            return (TextWidth <= FieldWidth);
        } else {
            return false;
        }
    };


    fontSize++;
    FontSize: while (true) {
        var text = "";
        fontSize--;
        var textHeight = AcroForm.internal.calculateFontSpace("3", fontSize + "px", font).height;
        var startY = (formObject.multiline) ? height - fontSize : (height - textHeight) / 2;
        startY += lineSpacing;
        var startX = -borderPadding;

        var lastX = startX, lastY = startY;
        var firstWordInLine = 0, lastWordInLine = 0;
        var lastLength = 0;

        var y = 0;
        if (fontSize == 0) {
            // In case, the Text doesn't fit at all
            fontSize = 12;
            text = "(...) Tj\n";
            text += "% Width of Text: " + AcroForm.internal.calculateFontSpace(text, "1px").width + ", FieldWidth:" + width + "\n";
            break;
        }

        lastLength = AcroForm.internal.calculateFontSpace(textSplit[0] + " ", fontSize + "px", font).width;

        var lastLine = "";
        var lineCount = 0;
        Line:
            for (var i in textSplit) {
                lastLine += textSplit[i] + " ";
                // Remove last blank
                lastLine = (lastLine.substr(lastLine.length - 1) == " ") ? lastLine.substr(0, lastLine.length - 1) : lastLine;
                var key = parseInt(i);
                lastLength = AcroForm.internal.calculateFontSpace(lastLine + " ", fontSize + "px", font).width;
                var nextLineIsSmaller = isSmallerThanWidth(key, lastLine, fontSize);
                var isLastWord = i >= textSplit.length - 1;
                if (nextLineIsSmaller && !isLastWord) {
                    lastLine += " ";
                    continue; // Line
                } else if (!nextLineIsSmaller && !isLastWord) {
                    if (!formObject.multiline) {
                        continue FontSize;
                    } else {
                        if (((textHeight + lineSpacing) * (lineCount + 2) + lineSpacing) > height) {
                            // If the Text is higher than the FieldObject
                            continue FontSize;
                        }
                        lastWordInLine = key;
                        // go on
                    }
                } else if (isLastWord) {
                    lastWordInLine = key;
                } else {
                    if (formObject.multiline && ((textHeight + lineSpacing) * (lineCount + 2) + lineSpacing) > height) {
                        // If the Text is higher than the FieldObject
                        continue FontSize;
                    }
                }

                var line = '';

                for (var x = firstWordInLine; x <= lastWordInLine; x++) {
                    line += textSplit[x] + ' ';
                }

                // Remove last blank
                line = (line.substr(line.length - 1) == " ") ? line.substr(0, line.length - 1) : line;
                //lastLength -= blankSpace.width;
                lastLength = AcroForm.internal.calculateFontSpace(line, fontSize + "px", font).width;

                // Calculate startX
                switch (formObject.Q) {
                    case 2: // Right justified
                        startX = (width - lastLength - borderPadding);
                        break;
                    case 1:// Q = 1 := Text-Alignment: Center
                        startX = (width - lastLength) / 2;
                        break;
                    case 0:
                    default:
                        startX = borderPadding;
                        break;
                }
                text += (startX) + ' ' + (lastY) + ' Td\n';
                text += '(' + line + ') Tj\n';
                // reset X in PDF
                text += (-startX) + ' 0 Td\n';

                // After a Line, adjust y position
                lastY = -(fontSize + lineSpacing);
                lastX = startX;

                // Reset for next iteration step
                lastLength = 0;
                firstWordInLine = lastWordInLine + 1;
                lineCount++;

                lastLine = "";
                continue Line;
            }
        break;
    }

    returnValue.text = text;
    returnValue.fontSize = fontSize;

    return returnValue;
};

AcroForm.internal.calculateAppearanceStream = function (formObject) {
    if (formObject.appearanceStreamContent) {
        // If appearanceStream is already set, use it
        return formObject.appearanceStreamContent;
    }

    if (!formObject.V && !formObject.DV) {
        return;
    }

    // else calculate it

    var stream = '';


    var text = formObject.V || formObject.DV;

    var calcRes = AcroForm.internal.calculateX(formObject, text);

    stream += '/Tx BMC\n' +
        'q\n' +
            //color + '\n' +
        '/F1 ' + calcRes.fontSize + ' Tf\n' +
            // Text Matrix
        '1 0 0 1 0 0 Tm\n';
    // Begin Text
    stream += 'BT\n';
    stream += calcRes.text;
    // End Text
    stream += 'ET\n';
    stream += 'Q\n' +
        'EMC\n';


    var appearanceStreamContent = new AcroForm.createFormXObject(formObject);

    appearanceStreamContent.stream = stream;


    var appearance = {
        N: {
            'Normal': appearanceStreamContent
        }
    };

    return appearanceStreamContent;
};

/*
 * Converts the Parameters from x,y,w,h to lowerLeftX, lowerLeftY, upperRightX, upperRightY
 * @param x
 * @param y
 * @param w
 * @param h
 * @returns {*[]}
 */
AcroForm.internal.calculateCoordinates = function (x, y, w, h) {
    var coordinates = {};

    if (this.internal) {
        function mmtopx(x) {
            return (x * this.internal.scaleFactor);
        }

        if (Array.isArray(x)) {
            x[0] = AcroForm.scale(x[0]);
            x[1] = AcroForm.scale(x[1]);
            x[2] = AcroForm.scale(x[2]);
            x[3] = AcroForm.scale(x[3]);

            coordinates.lowerLeft_X = x[0] || 0;
            coordinates.lowerLeft_Y = (mmtopx.call(this, this.internal.pageSize.height) - x[3] - x[1]) || 0;
            coordinates.upperRight_X = x[0] + x[2] || 0;
            coordinates.upperRight_Y = (mmtopx.call(this, this.internal.pageSize.height) - x[1]) || 0;
        } else {
            x = AcroForm.scale(x);
            y = AcroForm.scale(y);
            w = AcroForm.scale(w);
            h = AcroForm.scale(h);
            coordinates.lowerLeft_X = x || 0;
            coordinates.lowerLeft_Y = this.internal.pageSize.height - y || 0;
            coordinates.upperRight_X = x + w || 0;
            coordinates.upperRight_Y = this.internal.pageSize.height - y + h || 0;
        }
    } else {
        // old method, that is fallback, if we can't get the pageheight, the coordinate-system starts from lower left
        if (Array.isArray(x)) {
            coordinates.lowerLeft_X = x[0] || 0;
            coordinates.lowerLeft_Y = x[1] || 0;
            coordinates.upperRight_X = x[0] + x[2] || 0;
            coordinates.upperRight_Y = x[1] + x[3] || 0;
        } else {
            coordinates.lowerLeft_X = x || 0;
            coordinates.lowerLeft_Y = y || 0;
            coordinates.upperRight_X = x + w || 0;
            coordinates.upperRight_Y = y + h || 0;
        }
    }

    return [coordinates.lowerLeft_X, coordinates.lowerLeft_Y, coordinates.upperRight_X, coordinates.upperRight_Y];
};

AcroForm.internal.calculateColor = function (r, g, b) {
    var color = new Array(3);
    color.r = r | 0;
    color.g = g | 0;
    color.b = b | 0;
    return color;
};

AcroForm.internal.getBitPosition = function (variable, position) {
    variable = variable || 0;
    var bitMask = 1;
    bitMask = bitMask << (position - 1);
    return variable | bitMask;
};

AcroForm.internal.setBitPosition = function (variable, position, value) {
    variable = variable || 0;
    value = value || 1;

    var bitMask = 1;
    bitMask = bitMask << (position - 1);

    if (value == 1) {
        // Set the Bit to 1
        var variable = variable | bitMask;
    } else {
        // Set the Bit to 0
        var variable = variable & (~bitMask);
    }

    return variable;
};
```

  

Documentation generated by JSDoc 3.4.2 on Sun Oct 09 2016 11:08:27 GMT+0100 (BST) using the docdash theme.
